# SARM1 activation induces reversible mitochondrial dysfunction and can be prevented in human neurons by antisense oligonucleotides

**DOI:** 10.1016/j.nbd.2025.106986

**Published:** 2025-06-03

**Authors:** Andrea Loreto, Kaitlyn M.L. Cramb, Lucy A. McDermott, Christina Antoniou, Ilenia Cirilli, Maria Claudia Caiazza, Elisa Merlini, Peter Arthur-Farraj, W. Daniel du Preez, Elliot D. Mock, Hien T. Zhao, David L. Bennett, Giuseppe Orsomando, Michael P. Coleman, Richard Wade-Martins

**Affiliations:** a Neuroscience, School of Medical Sciences, Faculty of Medicine and Health, University of Sydney, Sydney, Australia; b Save Sight Institute, Faculty of Medicine and Health, University of Sydney, Sydney, Australia; c Charles Perkins Centre, Faculty of Medicine and Health, University of Sydney, Sydney, Australia; d Oxford Parkinson’s Disease Centre and Department of Physiology, Anatomy & Genetics, Kavli Institute for Nanoscience Discovery, University of Oxford, Dorothy Crowfoot Hodgkin Building, South Parks Road, Oxford OX1 3QU, UK; e John van Geest Centre for Brain Repair, Department of Clinical Neurosciences, University of Cambridge, Forvie Site, Robinson Way, CB2 0PY Cambridge, UK; f Nuffield Department of Clinical Neurosciences, University of Oxford, Oxford, UK; g Department of Clinical Sciences (DISCO), Section of Biochemistry, Polytechnic University of Marche, Via Ranieri 67, Ancona 60131, Italy; h Blizard Institute, Barts and London School of Medicine and Dentistry, Queen Mary University London, UK; i Neuroscience Drug Discovery, Ionis Pharmaceuticals, Inc.,2855 Gazelle Court, Carlsbad, CA 92010, USA

**Keywords:** SARM1, Vacor ASO, Nicotinamide Axon degeneration, Mitochondrial dysfunction, Neuroablative

## Abstract

SARM1 is a key regulator of a conserved program of axon degeneration increasingly linked to human neuro-degenerative diseases. Pathological SARM1 activation causes rapid NAD consumption, disrupting cellular homeostasis and leading to axon degeneration. In this study, we develop antisense oligonucleotides (ASOs) targeting human SARM1, demonstrating robust neuroprotection against morphological, metabolic, and mitochondrial impairment in human iPSC-derived dopamine neurons induced by the lethal neurotoxin vacor, a potent SARM1 activator. Furthermore, our findings reveal that axon fragmentation can be prevented, and mitochondrial dysfunction reversed using the NAD precursor nicotinamide, a form of vitamin B_3_, even after SARM1 activation has occurred, when neurons are already unhealthy. This research identifies ASOs as a promising therapeutic strategy to block SARM1, and provides an extensive characterisation and further mechanistic insights that demonstrate the reversibility of SARM1 toxicity in human neurons. It also identifies the SARM1 activator vacor as a specific and reversible neuroablative agent in human neurons.

## 1 Introduction

**Fig. 1:**
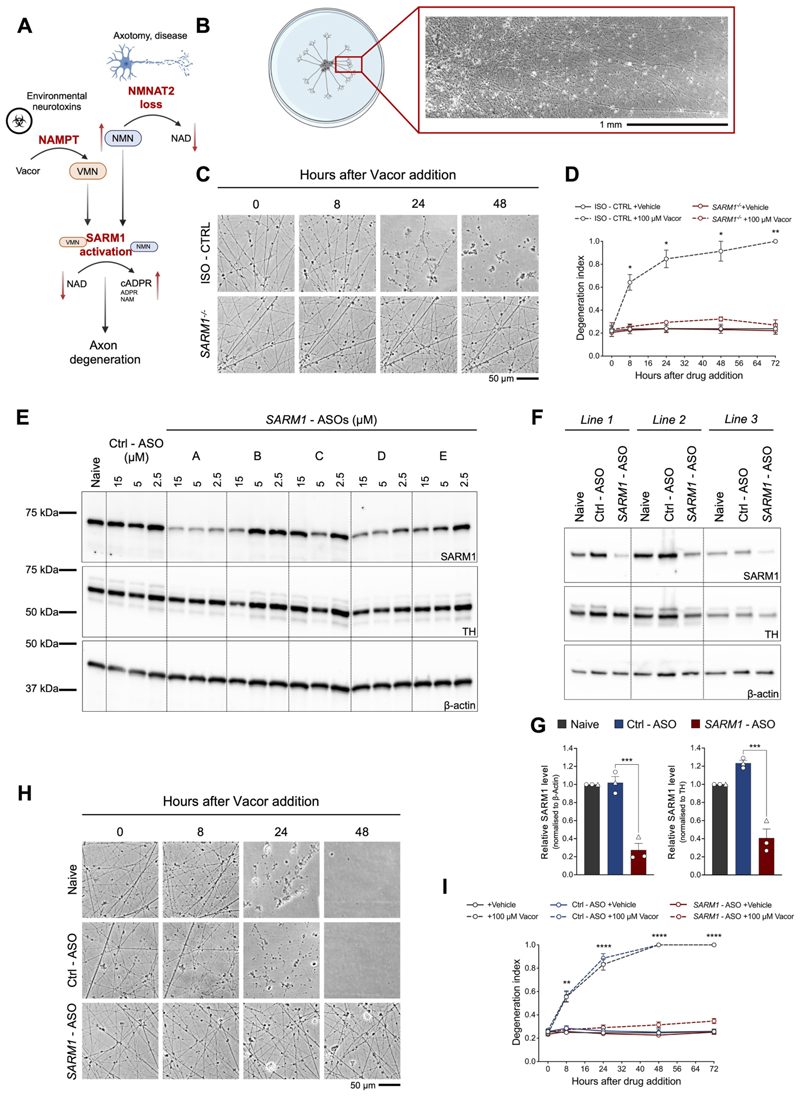
ASOs targeting human *SARM1* rescue human iPSC-derived dopamine neurons from vacor toxicity. (A) Schematic overview of programmed axon degeneration and the mechanism by which vacor acts on it. (B) Representative image of hiPSC-DANs plated as spot cultures growing long axons. (C) Representative images of axons from *SARM1^−/−^* and isogenic control (ISO - CTRL) hiPSC-DANs treated with vacor or vehicle. (D) Quantification of the degeneration index for the conditions described in part (C) (mean ± SEM; *n* = 3 from 3 independent differentiations; two-way RM ANOVA followed by Tukey’s multiple comparison test; statistical significance shown relative to *SARM1^−/−^* + 100 μM Vacor). (E) Representative immunoblots of hiPSC-DANs untreated (naive) or treated with *SARM1* - ASOs or a non-targeting control ASO (Ctrl - ASO) at the indicated concentrations, probed for SARM1, TH and β-actin (loading controls). (F) Representative immunoblots of hiPSC-DANs from 3 healthy individuals untreated, or treated with *SARM1* ‘A’ - ASO (this ASO was employed in all subsequent experiments at a concentration of 5 μM) or a non-targeting control ASO, probed for SARM1, TH and β-actin (loading controls) (*Line 1* = *067; Line 2* = *156; Line 3* = *053*). (G) Quantification of normalised SARM1 level (to β-actin and TH) is shown for the conditions described in part (F) (mean ± SEM; n = 3 from 1 independent differentiation; ordinary one-way ANOVA followed by Tukey’s multiple comparison test). (H) Representative images of axons from hiPSC-DANs untreated or treated with *SARM1* - ASO and Ctrl - ASO following administration of vacor or vehicle. (I) Quantification of the degeneration index for the conditions described in part (H) (mean ± SEM; *n* = 9 from 3 independent differentiations; two-way RM ANOVA followed by Tukey’s multiple comparison test; statistical comparison shown is Ctrl - ASO +100 μM Vacor vs *SARM1* - ASO +100 μM Vacor).

Sterile alpha and TIR motif-containing protein 1 (SARM1) is a central executor of programmed axon degeneration, an evolutionarily conserved pathway that is increasingly associated with human neuro-degenerative diseases (Coleman and Höke, 2020; Osterloh et al., 2012). SARM1 activity in neurons is tightly regulated by the axonal survival enzyme nicotinamide mononucleotide adenylyltransferase 2 (NMNAT2) (Gilley et al., 2015; Gilley and Coleman, 2010). Both SARM1 and NMNAT2 play a pivotal role in nicotinamide adenine dinucleotide (NAD) metabolism. Depletion or inactivation of NMNAT2 leads to an accumulation of its substrate nicotinamide mononucleotide (NMN), which then binds to SARM1, activating it (Di Stefano et al., 2017, 2015; Figley et al., 2021; Llobet Rosell et al., 2022; Loreto et al., 2023, 2015; Zhao et al., 2019). Once activated, SARM1 rapidly consumes NAD and causes axon degeneration (Essuman et al., 2017; Figley et al., 2021; Loreto et al., 2021). Vacor, a deadly environmental neurotoxin for humans (LeWitt, 1980), functions in a similar manner. Vacor is a pyridine derivative that is metabolised into vacor mononucleotide (VMN) and vacor adenine dinucleotide (VAD) by nicotinamide phosphoribosyltransferase (NAMPT) and NMNAT2 (Buonvicino et al., 2018). VMN, an analog of NMN, binds to and potently activates SARM1, causing neuron death (Loreto et al., 2021) (Fig. 1A). Vacor can also lead to inhibition of NAMPT and NMNAT2, which may further contribute to its toxic effect (Buonvicino et al., 2018). Other pyridine derivatives exhibit a similar SARM1-dependent neurotoxic mechanism (Angeletti et al., 2022; Huang et al., 2025; Wu et al., 2021). Importantly, pyridine scaffolds are widely used in pharmaceuticals, food, and cosmetics. Over 14 % of FDA-approved drugs contain a pyridine ring, many linked to neurotoxic side effects (Ling et al., 2021). This underscores the need to further investigate their potential role in human neurodegenerative diseases, possibly through SARM1-dependent mechanisms.

Targeting programmed axon degeneration holds significant potential for clinical applications (Krauss et al., 2020). *Sarm1* deletion is remarkably neuroprotective, as evidenced by the complete morphological and functional rescue of neurons in both genetic (Gilley et al., 2017) and toxic (Loreto et al., 2021) mouse models of neurodegeneration, each with human equivalents (LeWitt, 1980; Lukacs et al., 2019). Several preclinical studies suggest the involvement of the programmed axon degeneration pathway across a range of neurodegenerative disorders (Coleman and Höke, 2020). Notably, recent research has expanded upon findings derived from animal studies, identifying harmful gene variants of *SARM1* and *NMNAT2* in human disease. For instance, loss-of-function mutations in *NMNAT2* have been observed in patients with varying degrees of severity of polyneuropathy (Dingwall et al., 2022; Huppke et al., 2019; Lukacs et al., 2019). Genome-wide association studies (GWAS) have associated *SARM1* with amyotrophic lateral sclerosis (ALS) (Fogh et al., 2014; van Rheenen et al., 2016), and gain-of-function mutations in *SARM1* are notably prevalent in individuals affected by ALS and other motor neurone disorders (Bloom et al., 2022; Gilley et al., 2021).

To date, drug development has focused on inhibiting SARM1 activity through small molecule inhibitors (Bratkowski et al., 2022; Feldman et al., 2022; Hughes et al., 2021; Shi et al., 2022). Although these inhibitors are currently undergoing clinical trials, recent studies have reported unexpected toxicity at low doses when combined with mild SARM1 activation—such as exposure to a low, non-toxic dose of the SARM1 activator, vacor (Huang et al., 2025; Leahey et al., 2024; Wenbin et al., 2024). These findings underscore the need for diverse therapeutic strategies targeting SARM1, which can be used alone or in combination, to achieve the most effective therapeutic window and optimal neuroprotection. Among these approaches, antisense oligonucleotide (ASO) therapies hold significant promise. ASO-based treatments have already shown remarkable therapeutic effects in clinical settings for neurodegenerative disorders (Dhuri et al., 2020). Their mechanism of action is different from the currently in-development approaches targeting SARM1, as they lead to long-term reduction of the levels of the protein of interest rather than blocking its enzymatic activity (Roberts et al., 2020). We and others have recently demonstrated that ASOs against mouse *Sarm1* in rodent neurons confer neuroprotection (Gould et al., 2021; Liu et al., 2023). However, data in human neurons with ASOs targeting human *SARM1* are currently lacking.

In addition to developing effective therapies, essential questions remain about the mechanism of programmed axon degeneration. A deeper understanding of the molecular processes involved has revealed specific biomarkers of SARM1 activation and metabolic changes that occur before, or even in the absence, of axon degeneration and neuron loss (Antoniou et al., 2025; Li et al., 2021; Sasaki et al., 2020; Zhao et al., 2019). Notably, SARM1 activation lowers NAD levels and leads to cADPR accumulation, and how these metabolic changes affect cellular functionality remains unclear. Of particular interest is the connection between mitochondria and programmed axon degeneration. We and others have shown that mitochondrial dysfunction can both trigger and result from programmed axon degeneration activation (Loreto et al., 2020, 2015; Merlini et al., 2022; Summers et al., 2014). Nevertheless, it remains unclear whether SARM1 activation marks a critical point from which axons are inevitably committed to degeneration, with the sub-sequent mitochondrial dysfunction following SARM1 activation serving merely as a marker of degenerating neurons (Merlini et al., 2022). Linked to the concept of commitment to degeneration is the question of the reversibility of SARM1 toxicity and the associated functional alterations. For example, it is unclear whether morphological damage to axons and mitochondrial dysfunction downstream of SARM1 can be halted or even reversed once SARM1 activation has taken place. Understanding this is important not only for advancing our comprehension of programmed axon degeneration but also for potential clinical applications of therapies attempting to inhibit SARM1, considering that most diseases are diagnosed after pathological processes have been underway for some time. The question of reversibility is also relevant to therapies aiming to activate SARM1 for neuroablation to treat conditions such as spasticity, dystonia and neuropathic pain, because permanent SARM1 activation in these conditions could lead to unwanted effects and lasting disability. Lastly, while programmed axon degeneration has been extensively studied in animal models and rodent primary neuronal cultures, there is shortage of data from human neurons. Bridging this gap is essential as we progress toward clinical translation.

Here, we have developed ASOs targeting human *SARM1* and assessed their neuroprotective effects in human iPSC-derived dopamine neurons (hiPSC-DANs). Our findings reveal remarkable neuroprotection against morphological damage, metabolic changes, and mitochondrial dysfunction induced by the lethal neurotoxin and potent SARM1 activator vacor. Furthermore, we demonstrate that morphological damage to axons can be prevented, and mitochondrial dysfunction reversed using nicotinamide (NAM), a form of vitamin B_3_ and an NAD precursor, even hours after SARM1 activation has already occurred, when neurons are already unhealthy. This study indicates that toxicity caused by SARM1 activation in human neurons is reversible. The substantial neuroprotection achieved with *SARM1*-targeted ASOs in human neurons positions them as a promising therapeutic strategy to block or reverse morphological and functional damage caused by programmed axon degeneration activation. These findings also reveal the viability of small molecule SARM1 activators, such as vacor and its metabolite VMN, as selective and reversible neuroablative treatments in human neurons for neurological conditions such as spasticity, dystonia and neuropathic pain.

## 2 Materials and methods

### 2.1 Human iPSC-derived dopamine neuron production

Human induced pluripotent stem cells (hiPSCs) used in this study (Table 1) originated from the Oxford Parkinson’s Disease Center (OPDC) Discovery cohort. *SARM1*^−/−^ (knockout) iPSCs were generated using CRIPSR/Cas9. hiPSC-derived dopamine neurons were produced according to Protocols.io (Cramb et al., 2023b) as previously published (Williamson et al., 2023). In brief, hiPSCs were plated on Matrigel (BD Biosciences) and expanded in mTeSR1 medium (StemCell technologies) supplemented with mTeSR1 supplement (StemCell Technologies) and 100 U/ml Penicillin and 100 μg/ml Streptomycin (Life Technologies). Cells were passaged using EDTA or TrypLE (Life Technologies) and split 1:2 with 10 μM ROCK inhibitor (Y-27632) (Tocris Bioscience). Cells were differentiated into dopamine neurons using on a modified Krik’s protocol (Kriks et al., 2011), incorporating modifications adapted from (Fedele et al., 2017) as previously described (Williamson et al., 2023). hiPSCs were patterned to become ventral midbrain neural progenitors, expanded at this stage before being differentiated into dopamine neurons. hiPSC-DANs were quality controlled for differentiation efficiencies (Fig. S1D,E), half of the media was changed every 2–3 days and all experiments were performed between DIV 35 and 45. For spot cultures, 12-well plates were coated overnight with 1 % GelTrex (Thermo Fisher Scientific) diluted in H_2_O. The following day, GelTrex was removed and the well left to dry completely. This allowed to plate 10 μl ‘spots’ containing 100,000 hiPSC-DANs in culturing media at the centre of the well, minimising cell dispersion. Cells were given 30 min to attach in the incubator before the well was filled with 1 ml of culturing media.

### 2.2 Antisense oligonucleotides

The antisense oligonucleotides (ASOs) were developed and synthesised by Ionis Pharmaceuticals, as previously described (Swayze et al., 2007). The ASOs are 20 nucleotides in length, chemically modified MOE-gapmer oligonucleotides. The central gap segment comprises ten 2′-deoxyribonucleotides that are flanked on the 5′ and 3′ by five 2′MOE modified nucleotides. Internucleotide linkages are phosphorothioate, and all cytosine residues are 5′-methylcytosines. The sequences of the 5 ASOs targeting human *SARM1* are: ‘A’ - ASO 5′-CTACTTGTTTGTTAGTTCCA -3′; ‘B’ - ASO 5′-TCCACTATTTTTCCC-TACCT -3′, ‘C’ - ASO 5′-GTTGCTTTTCCTGTCATTAG -3′, ‘D’ - ASO 5′-GCACATATTTTATTTGCTAC -3′, ‘E’ - ASO 5′-GCAA-GATGTTTGCTTACCTG -3′ and a non-targeting Ctrl - ASO 5′-CCTA-TAGGACTATCCAGGAA -3′. ASO stock solutions were formulated at 15 mM in PBS (without CaCl_2_ and MgCl_2_) (Merck). ASOs were added to the culture media on DIV 22 and subsequently reapplied at every half media change. Therefore, the ASOs were present in the culture media for a minimum of 13 days before starting the experiments. Unless otherwise indicated, *SARM1* ‘A’ - ASO was employed for most experiments in this study at a final concentration of 5 μM.

### 2.3 Drug treatments and axotomy

hiPSC-DANs neurons were treated with vacor (Greyhound Chromatography) or vehicle (DMSO) just prior to imaging (time 0 h) or hiPSC-DANs cell pellets were collected in ice-cold PBS and were transected using a scalpel between DIV 35 and 45. When used, FK866 (kind gift of Prof Armando Genazzani, University of Piemonte Orientale) was added at the same time as vacor. When used, NAM was added at the same time or 2 and 4 h after vacor, as detailed in the results section and in (Fig. 4A; Fig. S5B). The drug concentration used is indicated in the figures and figure legends. Vacor was dissolved in DMSO; quantitation of the dissolved stock was performed spectrophotometrically (ε340 nm 17.8 mM^−1^ cm^−1^). FK866 was dissolved in DMSO and NAM was dissolved in UltraPure DNase/Rnase-free distilled water (Invitrogen).

### 2.4 Acquisition of phase contrast images and quantification of axon degeneration

Phase contrast images of hiPSC-DANs axons were acquired on a Nikon ECLIPSE TE2000-E upright fluorescence microscope coupled to a monochrome digital camera. For axon degeneration assays, a 20× objective was used and the degeneration index was determined using a Fiji plugin (Sasaki et al., 2009). For each experiment, the average was calculated from three fields per condition.

### 2.5 Metabolite analysis

Following treatment with ASOs, vacor and vehicle, hiPSC-DANs were washed in ice-cold PBS and rapidly frozen in dry ice and stored at −80 °C until processed. Tissues were extracted in HClO_4_ by sonication, followed by neutralisation with K_2_CO_3_. AMP, ADP, ATP and cADPR levels were measured by direct UV analysis by ion-pair C18-HPLC in the presence of tetrabutylammonium hydrogen sulfate. NMN and NAD levels were measured by spectrofluorometric HPLC analysis after derivatisation with acetophenone (Mori et al., 2014). Metabolite levels were normalised to protein levels quantified with the Bio-Rad Protein Assay (Bio-Rad) on formate-resuspended pellets from the HClO_4_ extraction.

### 2.6 Seahorse assay

hiPSC-DANs were plated in a XF96 Polystyrene Cell Culture Microplate (Seahorse Bioscience) at 50,000 cells/ well. Assay medium was prepared fresh the day of the assay using XF Base Medium (Agilent Technologies) with 10 mM Glucose (Merck), 1 mM Sodium Pyruvate (Merck) and 2 mM L-Glutamine (Thermo Fisher Scientific). Mitochondrial respiration was measured using a Seahorse Xfe96 Analyzer (Agilent Technologies). Three baseline recordings were taken followed by three recordings after each of the following subsequent injections: 1 μM Oligomycin (Merck), 1 μM FCCP (Merck) and 5 μM Rotenone and Antimycin (R/A) (Merck). Mitochondrial respiration was measured using a Seahorse Xfe96 Analyzer (Agilent Technologies) and normalised to basal oxygen consumption rate. For each experiment, the average was calculated from three to five wells per condition.

### 2.7 Western blot

hiPSC-DANs cell pellets were collected in ice-cold PBS and were homogenised in RIPA buffer containing protease (cOmplete EDTA-free, Roche) and phosphatase inhibitors (PhosSTOP, Roche). Protein concentration was determined by BCA assay (Thermofisher). 5 μg of sample were loaded on a 4-to-20 % SDS polyacrylamide gel (Bio-Rad). Membranes were blocked for 3 h in 5 % milk in TBS (50 mM Trizma base and 150 mM NaCl, PH 8.3, both Merck) plus 0.05 % Tween-20 (TBST) (Merck), incubated overnight with primary antibody in 5 % milk in TBST at 4 °C and subsequently washed in TBST and incubated for 1 h at room temperature with HRP-linked secondary antibody (Bio-Rad) in 5 % milk in TBST. Membranes were washed, treated with SuperSignal West Dura Extended Duration Substrate (Thermo Fisher Scientific) and imaged with BioRad imaging system. The following primary antibodies were used: rabbit anti-SARM1 (Cell Signaling Technology, 13022, 1:2,000), rabbit anti- tyrosine hydroxylase (TH) (Merck, AB152, 1:30,000) and mouse anti-β-actin (Merck, A5316, 1:50,000) as a loading control. Quantification of band intensity was determined by densitometry using Fiji.

### 2.8 Immunocytochemistry

hiPSC-DANs were plated on a 96-well plate, were fixed for 7 min using 4 % PFA, permeabilized with 0.1 % Triton-X in PBS, blocked for 1 h with 10 % serum in PBS and incubated overnight at 4 °C with the following primary antibodies in PBS: chicken anti-MAP2 (Abcam, ab92434, 1:500), rabbit anti- tyrosine hydroxylase (TH) (Merck, AB152, 1:30,000). Cells were then washed with PBS and incubated for 1 h at room with the following secondary antibodies: donkey anti-rabbit Alexafluor 488 and donkey anti-chicken Alexafluor 647 (both Thermo Fisher Scientific, A21206, A78952) washed one time with PBS and one time with PBS +DAPI (1:10 000) and subsequently imaged on the Opera Phenix High Content Screening System (Perkin Elmer) on a 40× water objective. Differentiation efficiency was quantified using ImageJ macro ‘cell counter’ for each differentiation using three pictures per well, three wells per line.

### 2.9 Statistical analysis

Graphs were produced using Prism GraphPad software as was the analysis to test for statistical significance (GraphPad Software, La Jolla, USA). A total of five hiPSC control lines derived from different healthy individuals were used throughout this study. For all experiments, except those involving the *SARM1^−/−^* line and its isogenic control, a minimum of three lines were differentiated in parallel and data originate from multiple, independent differentiations. The hiPSC lines used in each experiment are identifiable by a unique symbol (Table 1). The appropriate statistical test used, the n number (reflecting the total number of hiPSC lines used across multiple differentiations) and the number of differentiations for each experiment are indicated in the figure legends. A *p*-value <0.05 was considered significant. In figures, p-value >0.05 is reported as NS (not significant), p-value between 0.01 and 0.05 is reported as *, between 0.001 and 0.01 is reported as **, between 0.0001 and 0.001 Is reported as ***, and p-value <0.0001 is reported as ****.

## 3 Results

### 3.1 ASOs targeting human SARM1 rescue human iPSC-derived dopamine neurons from vacor toxicity

To assess axon degeneration in human neurons, we employed a spot culture technique to confine the cell bodies of hiPSC-DANs within a small area at the center of the cell culture dish. Fifteen days after the last replating, these spot-cultured hiPSC-DANs had developed long axons radiating outward, which was optimal for subsequent imaging and quantification of axon degeneration (Fig. 1B).

We have previously demonstrated that the neurotoxin vacor specifically activates SARM1 in mouse neurons, resulting in axon degeneration and neuronal soma death (Loreto et al., 2021). We first sought to establish whether vacor induces axon degeneration in human neurons through the same mechanism. To this end, we generated a *SARM1* knockout (*SARM1^−/−^*) hiPSC line using CRISPR/Cas9 gene editing. We validated the line by confirming the absence of detectable SARM1 protein following differentiation into dopamine neurons (Fig. S1A) and axon protection after axotomy (Chen et al., 2021; Osterloh et al., 2012) (Fig. S1B,C). *SARM1* deletion did not affect the efficiency of differentiation into dopamine neurons, as evidenced by the percentage of neurons expressing dopamine neuronal markers such as tyrosine hydroxylase (TH), which were comparable to those derived from the isogenic control line (ISO - CTRL) as well as other healthy individuals that were used throughout the study (Fig. S1D,E). We then tested whether vacor induces SARM1-dependent axon degeneration in human neurons. We observed dose-dependent axon degeneration in hiPSC-DANs (Fig. S2A,B) and, notably, complete prevention of vacor-induced axon degeneration was observed in *SARM1^−/−^* hiPSC-DANs (Fig. 1C,D). Consistent with findings in mouse neurons, vacor toxicity was also alleviated by inhibiting NAMPT with FK866, thereby blocking the conversion of vacor into the toxic metabolite and SARM1 activator VMN (Buonvicino et al., 2018; Loreto et al., 2021) (Fig. S2C,D). Thus, vacor requires SARM1 to cause axon degeneration in human neurons.

Next, we designed five ASOs that target human *SARM1* and evaluated their effectiveness in reducing SARM1 levels in hiPSC-DANs. All ASOs reduced SARM1 expression levels with variable efficiencies, as compared to both the non-targeting control ASO (Ctrl - ASO) and untreated hiPSC-DANs (Naive). *SARM1* ‘A’ - ASO demonstrated the most efficient knockdown (Fig. 1E) consistently across hiPSC-DAN lines from different healthy individuals (Fig. 1F,G) and robustly preserved axons from degeneration following vacor treatment (Fig. 1H,I). The strength of protection correlated with the efficiency of knockdown, as the less effective *SARM1* ‘D’ - ASO also provided axonal protection, albeit to a lesser extent (Fig. S3A,B). In contrast, the ineffective *SARM1* ‘C’ - ASO failed to protect axons following vacor administration (Fig. S3C,D). This correlation provides evidence that the protection against axon degeneration is linked to the ASOs’ ability to reduce SARM1 levels and does not result from off-target effects. *SARM1* ‘A’ - ASO was employed in all subsequent experiments. These findings indicate that ASOs targeting SARM1 are a viable approach to block programmed axon degeneration in human neurons.

### 3.2 ASOs targeting human SARM1 prevent metabolic changes and mitochondrial dysfunction caused by SARM1 activation

**Fig. 2:**
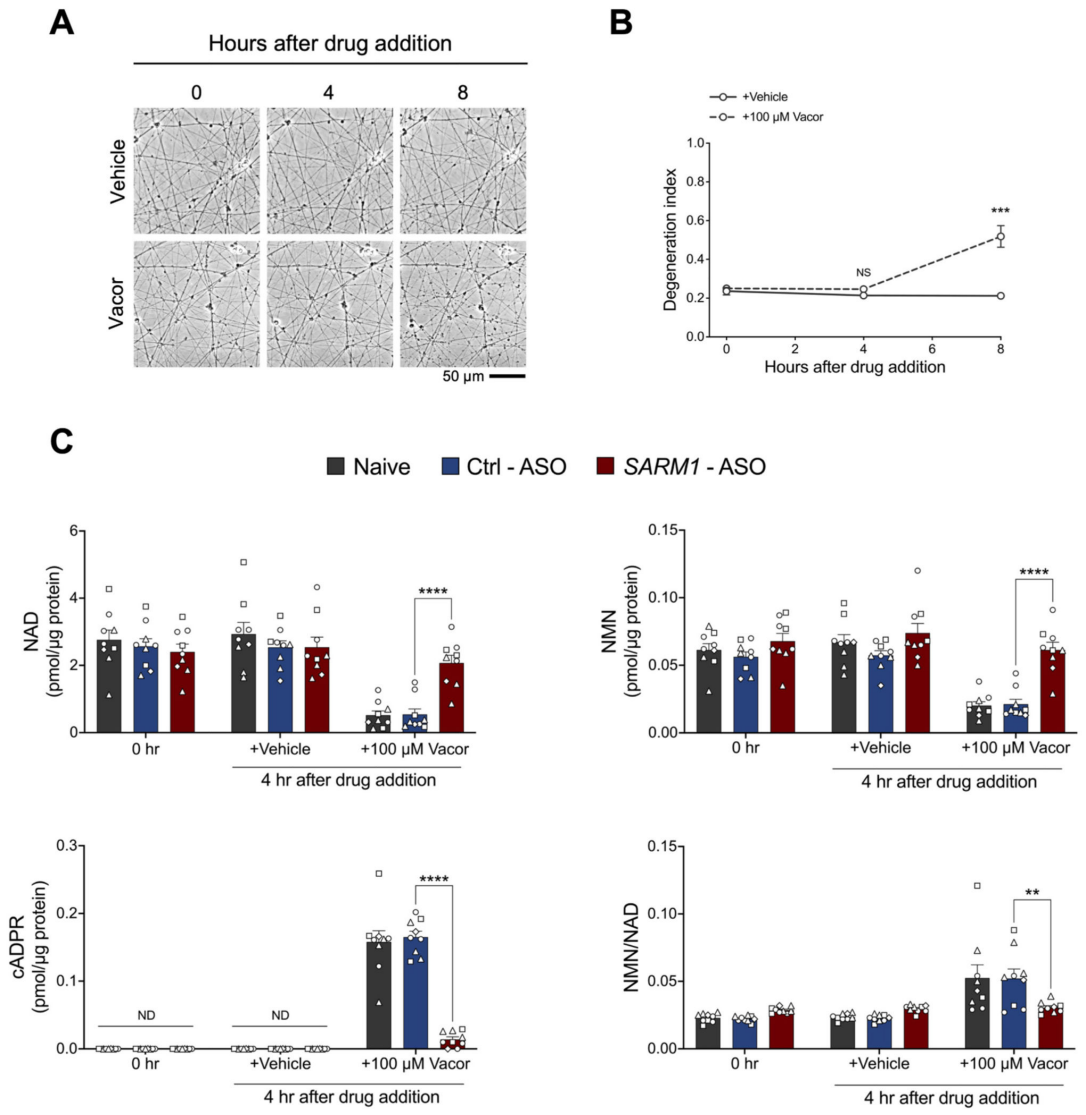
ASOs targeting human *SARM1* prevent metabolic changes caused by SARM1 activation. (A) Representative images of axons from hiPSC-DANs at the indicated time points after administration of vacor or vehicle. (B) Quantification of the degeneration index for the conditions described in part (A) (mean ± SEM; *n* = 11 from 3 independent differentiations; two-way RM ANOVA followed by Šídák’s multiple comparisons test). (C) NAD, cADPR, NMN levels and NMN/NAD ratio in hiPSC-DANs untreated (0 h) or treated with *SARM1* - ASO and Ctrl - ASO at the indicated time points following administration of vacor or vehicle (mean ± SEM; n = 9 from 3 independent differentiations; ordinary two-way ANOVA followed by Tukey’s multiple comparison test).

We next aimed to investigate whether ASOs targeting SARM1 could not only rescue morphological damage but also block SARM1-dependent metabolic changes that may impact cellular functionality. Active SARM1 rapidly consumes NAD and produces cADPR before observable axon degeneration and therefore, can serve as a marker specific for SARM1 activity in neurons (Loreto et al., 2021; Sasaki et al., 2020). We first tested if there are metabolic changes in vacor-treated hiPSC-DANs before axon degeneration was observable. We collected hiPSC-DANs 4 h after vacor treatment, at which point axons appeared morphologically unchanged (Fig. 2A,B), and we measured the metabolite levels. We found a marked decrease in NAD levels, along with an increase in cADPR levels and the NMN/NAD ratio. These metabolic changes were rescued by *SARM1* - ASO (Fig. 2C). Interestingly, and in line with findings in mouse neurons, NMN levels were also lowered in a SARM1-dependent manner (Loreto et al., 2021) (Fig. 2C).

**Fig. 3:**
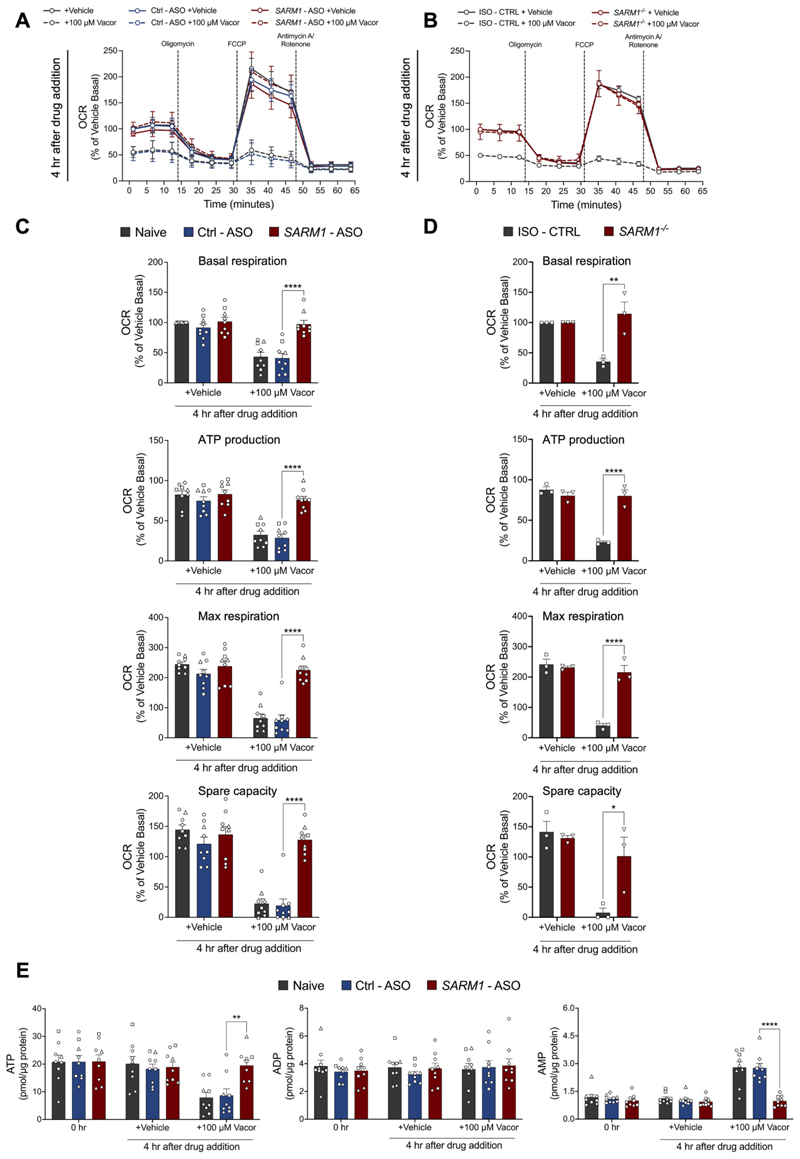
ASOs targeting human *SARM1* prevent mitochondrial dysfunction caused by SARM1 activation. (A,B) Mitochondrial respiration in hiPSC-DANs untreated, or treated with *SARM1* - ASO and Ctrl - ASO (A), and in *SARM1^−/−^* and ISO - CTRL hiPSC-DANs (B) at the indicated time point following administration of vacor or vehicle. Oxygen consumption rate (OCR) was normalised to basal respiration of vehicle treated hiPSC-DANs within each individual line and shown as a % change (mean ± SEM; n = 9 from 3 independent differentiations; ordinary two-way ANOVA followed by Tukey’s multiple comparison test). (C,D) Quantification of basal respiration, ATP production, maximal respiration and spare capacity for the conditions described in part (A) and (B). (E) ATP, ADP and AMP levels in hiPSC-DANs untreated (0 h), or treated with *SARM1* - ASO and Ctrl - ASO at the indicated time points following administration of vacor or vehicle (mean ± SEM; n = 9 from 3 independent differentiations; ordinary two-way ANOVA followed by Tukey’s multiple comparison test).

NAD is a key molecule for mitochondrial energy metabolism and mitochondrial dysfunction has been reported after programmed axon degeneration activation in mouse neurons (Merlini et al., 2022). We therefore evaluated whether specific SARM1 activation impairs mitochondrial respiration in hiPSC-DANs using the Seahorse Assay, and if this occurs prior to, or simultaneously alongside axon degeneration. In a time-course experiment of basal oxygen consumption rate (OCR), we observed that mitochondrial impairment began approximately 2 h after vacor treatment, indicating that mitochondrial respiration began to decline in a SARM1-dependent manner well before axon degeneration was detectable (Fig. S4A,B). We found that 4 h after vacor treatment, when no axon degeneration is observed (Fig. 2A,B) there was a dramatic, SARM1-dependent disruption in mitochondrial respiration resulting in impaired basal respiration, maximal respiration, ATP production, and spare capacity (Fig. 3A-D). Remarkably, mitochondrial respiration remained unaltered in *SARM1* - ASO treated hiPSC-DANs, matching the rescue observed with *SARM1* deletion (Fig. 3A-D). Furthermore, HPLC measurements revealed a substantial loss of ATP, and a concomitant increase in AMP level caused by vacor treatment, while ADP levels remained unchanged (Fig. 3E). This disruption was once again rescued by the application of *SARM1* - ASO (Fig. 3E). The lack of change in ADP levels is likely due to compensatory pathways, such as adenylate kinase activity, which converts excess ADP into ATP and AMP under stress to maintain energy homeostasis (Dzeja and Terzic, 2009).

Taken together, these data demonstrate that ASOs targeting *SARM1* effectively rescue both morphological axonal damage, metabolic changes and cellular functionality in human neurons following SARM1 activation. They also confirm that SARM1 activation is detectable before morphological damage is present and that mitochondrial dysfunction precedes axon degeneration in hiPSC-DANs after SARM1 activation, and this coincides with detectable metabolic changes.

### 3.3 Prevention of axon degeneration and improvement of mitochondrial dysfunction are achievable even after SARM1 activation has occurred

The increase in cADPR levels, the marked lowering of NAD levels and mitochondrial dysfunction indicate that SARM1 is active in hiPSC-DANs by 4 h of exposure to vacor when no observable axon degeneration is evident. The question remains whether SARM1 activation signifies an irreversible commitment to degeneration and whether changes in cellular functionality are, at least in part, a marker of degenerating neurons or an active process that can be reversed.

**Fig. 4:**
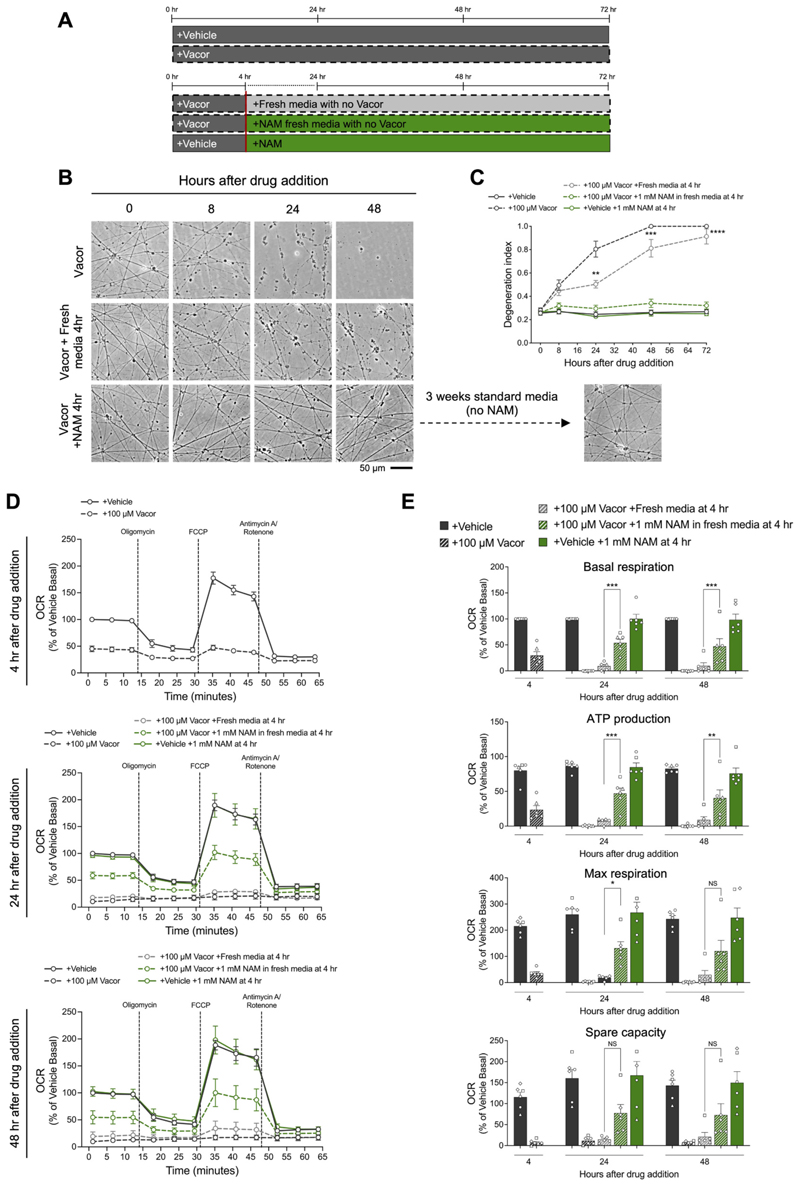
Prevention of axon degeneration and improvement of mitochondrial dysfunction are achievable even after SARM1 activation has occurred. (A) Schematic overview of the experimental design for experiments in (B-E). Fresh media with or without NAM was administered 4 h after vacor treatment while simultaneously removing vacor by replacing culture media. (B) Representative images of axons from hiPSC-DANs following treatments outlined in part (A). (C) Quantification of the degeneration index for the conditions described in part (B) (mean ± SEM; n = 9 from 3 independent differentiations; Mixed-effects model REML followed by Tukey’s multiple comparison test; statistical comparison shown is +100 μM Vacor +Fresh media at 4 h vs +100 μM Vacor +1 mM NAM in fresh media at 4 h). (D) Mitochondrial respiration in hiPSC-DANs following treatments outlined in part (A), at the indicated time point following drug administration. OCR was normalised to basal respiration of vehicle treated hiPSC-DANs within each individual line and shown as a % change. (E) Quantification of basal respiration, ATP production, maximal respiration and spare capacity for the conditions described in part (D) (mean ± SEM; *n* = 6 from 2 independent differentiations; ordinary two-way ANOVA followed by Tukey’s multiple comparison test).

To address this, we designed a series of experiments to determine if axon degeneration can be rescued and mitochondrial dysfunction reversed once SARM1 activation has already occurred. We have previously shown that NAM protects neurons against vacor toxicity by competing with vacor for NAMPT, thereby reducing the accumulation of the toxic vacor metabolite and the SARM1 activator VMN (Loreto et al., 2021). Additionally, NAM might also enhance NAD production and counteract NAD depletion downstream of SARM1 activation (Fig. S5A). We therefore hypothesised that NAM treatment could reverse vacor toxicity hours after vacor addition, when metabolite DANs and mitochondrial dysfunction were already evident. Initially, we treated hiPSC-with NAM either concurrently with vacor, or 2 and 4 h after vacor exposure changes (Fig. S5B). NAM treatment significantly delayed axon degeneration in hiPSC-DANs, whether it was added at the same time of vacor, or hours after (Fig. S5C,D). Although delayed, axon degeneration still occurred under these conditions over the following 72 h (Fig. S5C, D). To test whether vacor toxicity could be fully reversed, we adminis-tered NAM 4 h after vacor treatment while simultaneously removing vacor by replacing culture media with fresh media containing NAM (Fig. 4A). Remarkably, under these conditions axon degeneration was completely halted (Fig. 4B,C). In contrast, vacor removal and its replacement with fresh media without NAM marginally delayed but did not prevent degeneration (Fig. 4B,C). Three days after treatment with vacor and NAM, we replaced the media and reverted to standard media conditions without extra NAM addition, and the axons remained morphologically intact for the subsequent weeks (Fig. 4B), indicating a complete rescue of the morphological damage to axons.

Next, we sought to determine if mitochondrial dysfunction could be reversed after its onset, following SARM1 activation. As previously demonstrated, severe mitochondrial impairment was evident 4 h following vacor treatment. Using the same experimental paradigm of adding NAM and removing vacor at 4 h, we observed a significant improvement in mitochondrial functionality at both 24 and 48 h (Fig. 4D,E). Multiple mitochondrial parameters, including basal respiration, ATP production and maximal respiration were significantly improved compared to hiPSC-DANs that received fresh media without NAM at the 4-h mark (Fig. 4D,E). Moreover, we found a similar improving trend in spare capacity, although statistical significance was not reached (Fig. 4D,E).

Taken together, our experiments show that axon degeneration can still be rescued, and mitochondrial dysfunction can be improved, hours after SARM1 activation has taken place when metabolic changes and cellular dysfunction are already observable. These data demonstrate that SARM1 activation does not inevitably lead to axon degeneration, and its detrimental effects on cellular functionality can be reversed.

## 4 Discussion

In this study, we show that ASOs targeting human *SARM1* are a promising therapeutic approach to block programmed axon degeneration in human neurons. Our findings indicate that ASOs effectively delay morphological damage to axons and improve cellular functionality, including metabolic changes and mitochondrial dysfunction resulting from SARM1 activation. Notably, ASOs achieve this neuroprotective effect against vacor toxicity, a neurotoxin lethal to humans and currently the most potent known chemical SARM1 activator. These ASOs are also a valuable research tool due to their ease of use, being readily added to cell culture media. This makes them particularly advantageous in human iPSC models, enabling the study of treatment effects on the same cell line potentially reducing variability, a common limitation in iPSC models. Furthermore, mechanistic insights suggest that morphological damage can be prevented, and functional impairment can be reversed after SARM1 activation has occurred, which has important implications for clinical translation. Lastly, we provide an extensive characterisation of the consequences of SARM1 activation in human neurons, filling an important gap in the field. As we previously suggested (Loreto et al., 2021), vacor proves to be an excellent tool for drug discovery and can be used in large-scale drug screening programs targeting SARM1 in human neurons.

In recent years, ASO-based therapies have gained momentum, with several receiving FDA approval, thus highlighting their potential as a safe and effective therapeutic strategy. Notably, some of these therapies have yielded remarkable clinical outcomes (Dhuri et al., 2020). ASOs targeting human *SARM1* are part of a broader array of approaches under development to target SARM1, which also include small molecule inhibitors and gene therapy (Bosanac et al., 2021; Bratkowski et al., 2022; Feldman et al., 2022; Geisler et al., 2019; Hughes et al., 2021). Among these, a class of small molecule inhibitors targeting SARM1 catalytic site has been the most extensively developed, showing significant promise and already progressing to clinical trials (Bratkowski et al., 2022; Hughes et al., 2021). However, recent studies have raised concerns, suggesting that low doses of these inhibitors may paradoxically activate SARM1, leading to toxicity (Huang et al., 2025; Leahey et al., 2024; Wenbin et al., 2024). These findings underscore the likelihood that patient compliance with the drug regimen will be critical for therapeutic success. They also emphasise the importance of developing alternative strategies. ASOs that target human *SARM1* represent a strong alternative to small molecule inhibitors to achieve targeted and sustained SARM1 inhibition. In addition, having different therapeutic options is important for tailoring treatments to individual patients and maximising benefits while minimising potential risks. This is important because, while targeting SARM1 holds promise as a therapy, the physiological roles of SARM1 are still not fully understood, particularly in the context of potential side effects. Largely positive data from animal studies support the reduction in SARM1 as a favourable therapeutic target as SARM1-deficient animals generally maintain good health even into old age (Gilley et al., 2017). However, these animals are kept in highly controlled, pathogen-free environments, which could mask potential detrimental effects of SARM1 deficiency. For instance, several studies suggest that SARM1 may have important roles in preventing viral damage or spread (Crawford et al., 2022; Sundaramoorthy et al., 2020; Szretter et al., 2009). Other research also suggests a physiological role of SARM1 in regulating synaptic plasticity (Lin et al., 2014) and preventing local inflammation (Sun et al., 2021). Depending on the specific disease context and pathogenesis, localised ASO treatments may offer advantages over other putative SARM1-targeting therapies that act systemically, as they can help minimise off-target effects. As clinical trials targeting SARM1 are underway, there is a pressing need to address these considerations more directly.

Another significant finding from our study is that SARM1 activation induces dramatic cellular dysfunction, yet it does not signify a point of no return where axons are irreversibly committed to degeneration. We expand on previous research that, using biochemical assays, has suggested that SARM1 enzymatic activity is reversible (Zhao et al., 2019). While our study does not directly investigate the cellular reversibility of SARM1 enzymatic activity, it does provide evidence that axons can be preserved, and mitochondrial dysfunction can be improved after SARM1 activation is detected by metabolic changes and significant cellular dysfunction has already occurred. Prior to this work, the reversibility of SARM1 toxicity in human neurons had not been tested. This finding is critical for clinical translation considering that when most neurodegenerative diseases are diagnosed, after symptoms have already set in, cellular dysfunction such as mitochondrial impairment and axon degeneration are well underway (Cramb et al., 2023a). In future studies, it will be important to determine whether the reversal of SARM1 toxicity is specific to NAM treatment or if other NAD precursors, such as nicotinamide riboside, NMN, or SARM1 inhibitors, are also effective. It will also be important to include measurements of electrophysiological activity and neurotransmitter release to better define the extent of functional rescue. The observed improvement in mitochondrial function suggests that, to some extent, mitochondrial dysfunction is linked to metabolic changes triggered by SARM1 activation, rather than being caused by irreversible organelle damage. The dramatic loss of NAD due to SARM1 activation likely significantly contributes to mitochondrial respiration failure. These findings align with recent studies that demonstrate a role for SARM1 in exacerbating mitochondrial dysfunction in a model of Charcot-Marie-Tooth disease type 2 A (CMT2A) (Sato-Yamada et al., 2022), which emphasises the importance of further investigating the impact of SARM1 on mitochondrial function.

Distinct from strategies inhibiting SARM1 to protect neurons, this study also identifies pyridine derivatives that act as direct SARM1 activators, such as vacor and its metabolite VMN, as viable and reversible neuroablative agents in human neurons. We and others have previously shown that SARM1 activators cause specific neurodegeneration in mouse neurons (Loreto et al., 2021; Wu et al., 2021), and it had been postulated by Wu and colleagues that these could be used as neuro-ablative agents in certain conditions (Wu et al., 2021). However, until recently, it had not been known whether SARM1 was exclusively expressed in neurons, which would limit the use of SARM1 activators for selective neuroablation. Interestingly, it has recently been shown that oligodendrocytes, the myelinating glia in the central nervous system, contain high levels of SARM1 protein and are very sensitive to SARM1 activators (Fazal et al., 2023; Zeng et al., 2024). SARM1 has also been detected in murine astrocytes (Liu et al., 2021). However, Schwann cells, the glia of the peripheral nervous system, contain no functionally significant SARM1 protein and are completely insensitive to vacor and other SARM1 activators (Fazal et al., 2023). The combination of these findings with our data showing that SARM1 activation in human neurons is reversible raises the possibility that highly specific SARM1 activators could be peripherally administered for selective and reversible neuroablation in neurological conditions characterised by dystonia, spasticity, and neuropathic pain.

Lastly, while our current study employs vacor, which induces rapid axon degeneration, our findings with NAM strongly suggest that the cellular dysfunction caused by SARM1 activation can be dissociated from the morphological damage to axons. This distinction is pivotal, as it indicates that SARM1 activation can cause significant neuronal dysfunction without progressing to full-scale degeneration. Recent research has shown that SARM1 activation can occur at sublethal levels across various cell types (Zhao et al., 2019), including neurons (Antoniou et al., 2025; Li et al., 2021; Sasaki et al., 2020). However, the consequences of this sublethal activation on broader cellular function have remained largely unexplored. Our study suggests that SARM1 activation significantly affects cellular function even in the absence of axon degeneration in human neurons. Future studies should include experiments assessing the impact of programmed axon degeneration pathway activation on cellular functionality, especially in models where axon degeneration is not observed. This is relevant to numerous pre-clinical neurodegenerative disease models, especially in human iPSC-derived neuron studies. For instance, specifically most hiPSC-DANs lines derived from Parkinson’s disease (PD) patients do not exhibit clear morphological changes. This approach may unexpectedly reveal a broader involvement of programmed axon degeneration in chronic, slow-progressing neurodegenerative disorders, where dysfunction frequently precedes degeneration (Cramb et al., 2023a). To further strengthen disease relevance, future work could incorporate more acute, PD-relevant models, such as administration of α-synuclein pre-formed fibrils or mitochondrial toxins like 1-methyl-4-phenylpyridinium (MPP^+^) and rotenone.

In conclusion, we have comprehensively characterised the detrimental effects of SARM1 activation in human neurons, developed ASOs targeting human SARM1, and demonstrated their effective mitigation of morphological damage and cellular dysfunction. We have demonstrated the reversibility of SARM1 toxicity in human neurons and discussed its significance for clinical applications, both for therapies aiming at inhibiting or activating SARM1. Finally, we highlight the importance of expanding beyond morphology to consider functional impairments, especially in the context of sublethal SARM1 activation. This may reveal key insights for understanding the role of programmed axon degeneration in slow-progressing, chronic neurodegenerative diseases.

## Supplementary Material

Supplementary Material

## Figures and Tables

**Table 1:** Human iPSC lines used in this study.

Donor ID & Symbol	Genotype	Age & Gender	Isogenic Control
⬡JR053–1 (Lang et al., 2019)	Healthy control	68 M	–
◊SFC065–03–03 (Beccano-Kelly et al., 2023)	Healthy control	65 M	–
○SFC067–03–01 (Lang et al., 2019)	Healthy control	72 M	–
ΔSFC156–03–01 (Lang et al., 2019)	Healthy control	75 M	–
□SFCAD2–01 (Buskin et al., 2018) (SBAd2–01)	Healthy control (ISO - CTRL)	51 M	Parental background for AD2 - *SARM1*^−/−^
∇AD2 - *SARM1*^−/−^	*SARMr* ^−/−^	51 M	Isogenic null line from SFCAD2-01

## Data Availability

Data will be made available on request.

## References

[R1] Angeletti C, Amici A, Gilley J, Loreto A, Trapanotto AG, Antoniou C, Merlini E, Coleman MP, Orsomando G (2022). SARM1 is a multi-functional NAD(P)ase with prominent base exchange activity, all regulated bymultiple physiologically relevant NAD metabolites. iScience.

[R2] Antoniou C, Loreto A, Gilley J, Merlini E, Orsomando G, Coleman MP (2025). Chronically low NMNAT2 expression causes sub-lethal SARM1 activation and altered response to nicotinamide riboside in axons. Mol Neurobiol.

[R3] Beccano-Kelly DA, Cherubini M, Mousba Y, Cramb KML, Giussani S, Caiazza MC, Rai P, Vingill S, Bengoa-Vergniory N, Ng B, Corda G (2023). Calcium dysregulation combined with mitochondrial failure and electrophysiological maturity converge in Parkinson’s iPSC-dopamine neurons. iScience.

[R4] Bloom AJ, Mao X, Strickland A, Sasaki Y, Milbrandt J, DiAntonio A (2022). Constitutively active SARM1 variants that induce neuropathy are enriched in ALS patients. Mol Neurodegener.

[R5] Bosanac T, Hughes RO, Engber T, Devraj R, Brearley A, Danker K, Young K, Kopatz J, Hermann M, Berthemy A, Boyce S (2021). Pharmacological SARM1 inhibition protects axon structure and function in paclitaxel-induced peripheral neuropathy. Brain.

[R6] Bratkowski M, Burdett TC, Danao J, Wang X, Mathur P, Gu W, Beckstead JA, Talreja S, Yang Y-S, Danko G, Park JH (2022). Uncompetitive, adduct-forming SARM1 inhibitors are neuroprotective in preclinical models of nerve injury and disease. Neuron.

[R7] Buonvicino D, Mazzola F, Zamporlini F, Resta F, Ranieri G, Camaioni E, Muzzi M, Zecchi R, Pieraccini G, Dölle C, Calamante M (2018). Identification of the nicotinamide salvage pathway as a new Toxification route for antimetabolites. Cell Chem Biol.

[R8] Buskin A, Zhu L, Chichagova V, Basu B, Mozaffari-Jovin S, Dolan D, Droop A, Collin J, Bronstein R, Mehrotra S, Farkas M (2018). Disrupted alternative splicing for genes implicated in splicing and ciliogenesis causes PRPF31 retinitis pigmentosa. Nat Commun.

[R9] Chen Y-H, Sasaki Y, DiAntonio A, Milbrandt J (2021). SARM1 is required in human derived sensory neurons for injury-induced and neurotoxic axon degeneration. Exp Neurol.

[R10] Coleman MP, Höke A (2020). Programmed axon degeneration: from mouse to mechanism to medicine. Nat Rev Neurosci.

[R11] Cramb KML, Beccano-Kelly D, Cragg SJ, Wade-Martins R (2023a). Impaired dopamine release in Parkinson’s disease. Brain.

[R12] Cramb Kaitlyn, Malpartida AB, Caiazza MC, Wade-Martins R, Ryan B (2023b). Differentiation of Human Dopamine Neurons (DaNs) from Induced Pluripotent Stem Cells (iPSCs).

[R13] Crawford CL, Antoniou C, Komarek L, Schultz V, Donald CL, Montague P, Barnett SC, Linington C, Willison HJ, Kohl A, Coleman MP (2022). SARM1 depletion slows axon degeneration in a CNS model of neurotropic viral infection. Frontiers in Molecular Neuroscience.

[R14] Dhuri K, Bechtold C, Quijano E, Pham H, Gupta A, Vikram A, Bahal R (2020). Antisense oligonucleotides: an emerging area in drug discovery and development. J Clin Med.

[R15] Di Stefano M, Nascimento-Ferreira I, Orsomando G, Mori V, Gilley J, Brown R, Janeckova L, Vargas ME, Worrell LA, Loreto A, Tickle J (2015). A rise in NAD precursor nicotinamide mononucleotide (NMN) after injury promotes axon degeneration. Cell Death Differ.

[R16] Di Stefano M, Loreto A, Orsomando G, Mori V, Zamporlini F, Hulse RP, Webster J, Donaldson LF, Gering M, Raffaelli N, Coleman MP (2017). NMN Deamidase delays Wallerian degeneration and rescues axonal defects caused by NMNAT2 deficiency in vivo. Curr Biol.

[R17] Dingwall CB, Strickland A, Yum SW, Yim AK, Zhu J, Wang PL, Yamada Y, Schmidt RE, Sasaki Y, Bloom AJ, DiAntonio A (2022). Macrophage Depletion Blocks Congenital SARM1-Dependent Neuropathy.

[R18] Dzeja P, Terzic A (2009). Adenylate kinase and AMP signaling networks: metabolic monitoring, signal communication and body energy sensing. Int J Mol Sci.

[R19] Essuman K, Summers DW, Sasaki Y, Mao X, DiAntonio A, Milbrandt J (2017). The SARM1 toll/Interleukin-1 receptor domain possesses intrinsic NAD + cleavage activity that promotes pathological axonal degeneration. Neuron.

[R20] Fazal SV, Mutschler C, Chen CZ, Turmaine M, Chen C-Y, Hsueh Y-P, Ibañez-Grau A, Loreto A, Casillas-Bajo A, Cabedo H, Franklin RJM (2023). SARM1 detection in myelinating glia: sarm1/Sarm1 is dispensable for PNS and CNS myelination in zebrafish and mice. Front Cell Neurosci.

[R21] Fedele S, Collo G, Behr K, Bischofberger J, Müller S, Kunath T, Christensen K, Gündner AL, Graf M, Jagasia R, Taylor V (2017). Expansion of human midbrain floor plate progenitors from induced pluripotent stem cells increases dopaminergic neuron differentiation potential. Sci Rep.

[R22] Feldman HC, Merlini E, Guijas C, DeMeester KE, Njomen E, Kozina EM, Yokoyama M, Vinogradova E, Reardon HT, Melillo B, Schreiber SL (2022). Selective inhibitors of SARM1 targeting an allosteric cysteine in the autoregulatory ARM domain. Proc Natl Acad Sci.

[R23] Figley MD, Gu W, Nanson JD, Shi Y, Sasaki Y, Cunnea K, Malde AK, Jia X, Luo Z, Saikot FK, Mosaiab T (2021). SARM1 is a metabolic sensor activated by an increased NMN/NAD + ratio to trigger axon degeneration. Neuron.

[R24] Fogh I, Ratti A, Gellera C, Lin K, Tiloca C, Moskvina V, Corrado L, Sorarù G, Cereda C, Corti S, Gentilini D (2014). A genome-wide association meta-analysis identifies a novel locus at 17q11.2 associated with sporadic amyotrophic lateral sclerosis. Hum Mol Genet.

[R25] Geisler S, Huang SX, Strickland A, Doan RA, Summers DW, Mao X, Park J, DiAntonio A, Milbrandt J (2019). Gene therapy targeting SARM1 blocks pathological axon degeneration in mice. J Exp Med.

[R26] Gilley J, Coleman MP (2010). Endogenous Nmnat2 is an essential survival factor for maintenance of healthy axons. PLoS Biol.

[R27] Gilley J, Orsomando G, Nascimento-Ferreira I, Coleman MP (2015). Absence of SARM1 rescues development and survival of NMNAT2-deficient axons. Cell Rep.

[R28] Gilley J, Ribchester RR, Coleman MP (2017). Sarm1 deletion, but not WldS, confers lifelong rescue in a mouse model of severe axonopathy. Cell Rep.

[R29] Gilley J, Jackson O, Pipis M, Estiar MA, Al-Chalabi A, Danzi MC, van Eijk KR, Goutman SA, Harms MB, Houlden H, Iacoangeli A (2021). Enrichment of SARM1 alleles encoding variants with constitutively hyperactive NADase in patients with ALS and other motor nerve disorders. eLife.

[R30] Gould SA, Gilley J, Ling K, Jafar-Nejad P, Rigo F, Coleman M (2021). Sarm1 haploinsufficiency or low expression levels after antisense oligonucleotides delay programmed axon degeneration. Cell Rep.

[R31] Huang Y, Zhang J, Zhang W, Chen J, Chen S, Wu Q, Zheng S, Wang X (2025). Stepwise activation of SARM1 for cell death and axon degeneration revealed by a biosynthetic NMN mimic. Proc Natl Acad Sci.

[R32] Hughes RO, Bosanac T, Mao X, Engber TM, DiAntonio A, Milbrandt J, Devraj R, Krauss R (2021). Small molecule SARM1 inhibitors recapitulate the SARM1 -/- phenotype and allow recovery of a metastable Pool of axons fated to degenerate. Cell Rep.

[R33] Huppke P, Wegener E, Gilley J, Angeletti C, Kurth I, Drenth JPH, Stadelmann C, Barrantes-Freer A, Brück W, Thiele H, Nürnberg P (2019). Homozygous NMNAT2 mutation in sisters with polyneuropathy and erythromelalgia. Exp Neurol.

[R34] Krauss R, Bosanac T, Devraj R, Engber T, Hughes RO (2020). Axons matter: the promise of treating neurodegenerative disorders by targeting SARM1-mediated axonal degeneration. Trends Pharmacol Sci.

[R35] Kriks S, Shim J-W, Piao J, Ganat YM, Wakeman DR, Xie Z, Carrillo-Reid L, Auyeung G, Antonacci C, Buch A, Yang L (2011). Dopamine neurons derived from human ES cells efficiently engraft in animal models of Parkinson’s disease. Nature.

[R36] Lang C, Campbell KR, Ryan BJ, Carling P, Attar M, Vowles J, Perestenko OV, Bowden R, Baig F, Kasten M, Hu MT (2019). Single-cell sequencing of iPSC-dopamine neurons reconstructs disease progression and identifies HDAC4 as a regulator of Parkinson cell phenotypes. Cell Stem Cell.

[R37] Leahey R, Weber M, Cho CH, Hur S, Cramer A, Manzanares K, Babin B, Boenig G, Kring T, Liu L, Cui Y (2024). SARM1 Orthosteric Base Exchange Inhibitors Cause Subinhibitory SARM1 Activation.

[R38] LeWitt PA (1980). The neurotoxicity of the rat poison vacor. N Engl J Med.

[R39] Li WH, Huang K, Cai Y, Wang QW, Zhu WJ, Hou YN, Wang S, Cao S, Zhao ZY, Xie XJ, Du Y (2021). Permeant fluorescent probes visualize the activation of SARM1 and uncover an anti-neurodegenerative drug candidate. eLife.

[R40] Lin C-W, Chen C-Y, Cheng S-J, Hu H-T, Hsueh Y-P (2014). Sarm1 deficiency impairs synaptic function and leads to behavioral deficits, which can be ameliorated by an mGluR allosteric modulator. Front Cell Neurosci.

[R41] Ling Y, Hao Z-Y, Liang D, Zhang C-L, Liu Y-F, Wang Y (2021). The expanding role of pyridine and Dihydropyridine scaffolds in drug design. Drug Des Devel Ther.

[R42] Liu H, Zhang J, Xu X, Lu S, Yang D, Xie C, Jia M, Zhang W, Jin L, Wang X, Shen X (2021). SARM1 promotes neuroinflammation and inhibits neural regeneration after spinal cord injury through NF-κB signaling. Theranostics.

[R43] Liu P, Chen W, Jiang H, Huang H, Liu L, Fang F, Li L, Feng X, Liu D, Dalal R, Sun Y (2023). Differential effects of SARM1 inhibition in traumatic glaucoma and EAE optic neuropathies. Molecular Therapy - Nucleic Acids.

[R44] Llobet Rosell A, Paglione M, Gilley J, Kocia M, Perillo G, Gasparrini M, Cialabrini L, Raffaelli N, Angeletti C, Orsomando G, Wu P-H (2022). The NAD + precursor NMN activates dSarm to trigger axon degeneration in Drosophila. eLife.

[R45] Loreto A, Di Stefano M, Gering M, Conforti L (2015). Wallerian degeneration is executed by an NMN-SARM1-dependent late Ca2+influx but only modestly influenced by mitochondria. Cell Rep.

[R46] Loreto A, Hill CS, Hewitt VL, Orsomando G, Angeletti C, Gilley J, Lucci C, Sanchez-Martinez A, Whitworth AJ, Conforti L, Dajas-Bailador F (2020). Mitochondrial impairment activates the Wallerian pathway through depletion of NMNAT2 leading to SARM1-dependent axon degeneration. Neurobiol Dis.

[R47] Loreto A, Angeletti C, Gu W, Osborne A, Nieuwenhuis B, Gilley J, Arthur-Farraj P, Merlini E, Amici A, Luo Z, Hartley-Tassell L (2021). Neurotoxin-mediated --potent activation of the axon degeneration regulator SARM1. eLife.

[R48] Loreto A, Antoniou C, Merlini E, Gilley J, Coleman MP (2023). NMN: the NAD precursor at the intersection between axon degeneration and anti-ageing therapies. Neurosci Res.

[R49] Lukacs M, Gilley J, Zhu Y, Orsomando G, Angeletti C, Liu J, Yang X, Park J, Hopkin RJ, Coleman MP, Zhai RG (2019). Severe biallelic loss-of-function mutations in nicotinamide mononucleotide adenylyltransferase 2 (NMNAT2) in two fetuses with fetal akinesia deformation sequence. Exp Neurol.

[R50] Merlini E, Coleman MP, Loreto A (2022). Mitochondrial dysfunction as a trigger of programmed axon death. Trends Neurosci.

[R51] Mori V, Amici A, Mazzola F, Stefano MD, Conforti L, Magni G, Ruggieri S, Raffaelli N, Orsomando G (2014). Metabolic profiling of alternative NAD biosynthetic routes in mouse tissues. PloS One.

[R52] Osterloh JM, Yang J, Rooney TM, Fox AN, Adalbert R, Powell EH, Sheehan AE, Avery MA, Hackett R, Logan MA, MacDonald JM (2012). dSarm/Sarm1 is required for activation of an injury-induced axon death pathway. Science.

[R53] van Rheenen W, Shatunov A, Dekker AM, McLaughlin RL, Diekstra FP, Pulit SL, van der Spek RAA, Võsa U, de Jong S, Robinson MR, Yang J (2016). Genome-wide association analyses identify new risk variants and the genetic architecture of amyotrophic lateral sclerosis. Nat Genet.

[R54] Roberts TC, Langer R, Wood MJA (2020). Advances in oligonucleotide drug delivery. Nat Rev Drug Discov.

[R55] Sasaki Y, Vohra BPS, Lund FE, Milbrandt J (2009). Nicotinamide mononucleotide adenylyl transferase-mediated axonal protection requires enzymatic activity but not increased levels of neuronal nicotinamide adenine dinucleotide. J Neurosci.

[R56] Sasaki Y, Engber TM, Hughes RO, Figley MD, Wu T, Bosanac T, Devraj R, Milbrandt J, Krauss R, DiAntonio A (2020). cADPR is a gene dosage-sensitive biomarker of SARM1 activity in healthy, compromised, and degenerating axons. Exp Neurol.

[R57] Sato-Yamada Y, Strickland A, Sasaki Y, Bloom J, DiAntonio A, Milbrandt J (2022). A SARM1-mitochondrial feedback loop drives neuropathogenesis in a Charcot-Marie-tooth disease type 2A rat model. J Clin Invest.

[R58] Shi Y, Kerry PS, Nanson JD, Bosanac T, Sasaki Y, Krauss R, Saikot FK, Adams SE, Mosaiab T, Masic V, Mao X (2022). Structural basis of SARM1 activation, substrate recognition, and inhibition by small molecules. Mol Cell.

[R59] Summers DW, DiAntonio A, Milbrandt J (2014). Mitochondrial dysfunction induces Sarm1-dependent cell death in sensory neurons. J Neurosci.

[R60] Sun Y, Wang Q, Wang Y, Ren W, Cao Y, Li J, Zhou X, Fu W, Yang J (2021). Sarm1-mediated neurodegeneration within the enteric nervous system protects against local inflammation of the colon. Protein Cell.

[R61] Sundaramoorthy V, Green D, Locke K, O’Brien CM, Dearnley M, Bingham J (2020). Novel role of SARM1 mediated axonal degeneration in the pathogenesis of rabies. PLoS Pathog.

[R62] Swayze EE, Siwkowski AM, Wancewicz EV, Migawa MT, Wyrzykiewicz TK, Hung G, Monia BP, Bennett CF (2007). Antisense oligonucleotides containing locked nucleic acid improve potency but cause significant hepatotoxicity in animals. Nucleic Acids Res.

[R63] Szretter KJ, Samuel MA, Gilfillan S, Fuchs A, Colonna M, Diamond MS (2009). The immune adaptor molecule SARM modulates tumor necrosis factor alpha production and microglia activation in the brainstem and restricts West Nile virus pathogenesis. J Virol.

[R64] Wenbin Z, Qinyi Z, Jun Z, Jiachen W, Sanduo Z, Xiaodong W (2024). SARM1 Activation Promotes Axonal Degeneration Via a Two-Step Liquid-to-Solid Phase Transition.

[R65] Williamson MG, Madureira M, McGuinness W, Heon-Roberts R, Mock ED, Naidoo K, Cramb KML, Caiazza M-C, Malpartida AB, Lavelle M, Savory K (2023). Mitochondrial dysfunction and mitophagy defects in LRRK2-R1441C Parkinson’s disease models. Hum Mol Genet.

[R66] Wu T, Zhu J, Strickland A, Ko KW, Sasaki Y, Dingwall CB, Yamada Y, Figley MD, Mao X, Neiner A, Bloom AJ (2021). Neurotoxins subvert the allosteric activation mechanism of SARM1 to induce neuronal loss. Cell Rep.

[R67] Zeng H, Mayberry JE, Wadkins D, Chen N, Summers DW, Kuehn MH (2024). Loss of Sarm1 reduces retinal ganglion cell loss in chronic glaucoma. Acta Neuropathol Commun.

[R68] Zhao ZY, Xie XJ, Li WH, Liu J, Chen Z, Zhang B, Li T, Li SL, Lu JG, Zhang Liangren, Zhang Li-he (2019). A Cell-permeant mimetic of NMN activates SARM1 to produce cyclic ADP-Ribose and induce non-apoptotic Cell Death. iScience.

