# Supplementary material for: SARM1 activation induces reversible mitochondrial dysfunction and can be prevented in human neurons by antisense oligonucleotides
Source: Neurobiol Dis. Author manuscript; Available in PMC 2025 Jul 21. (PMC7617922; doi:10.1016/j.nbd.2025.106986)

Actin Fig 1E and S1A

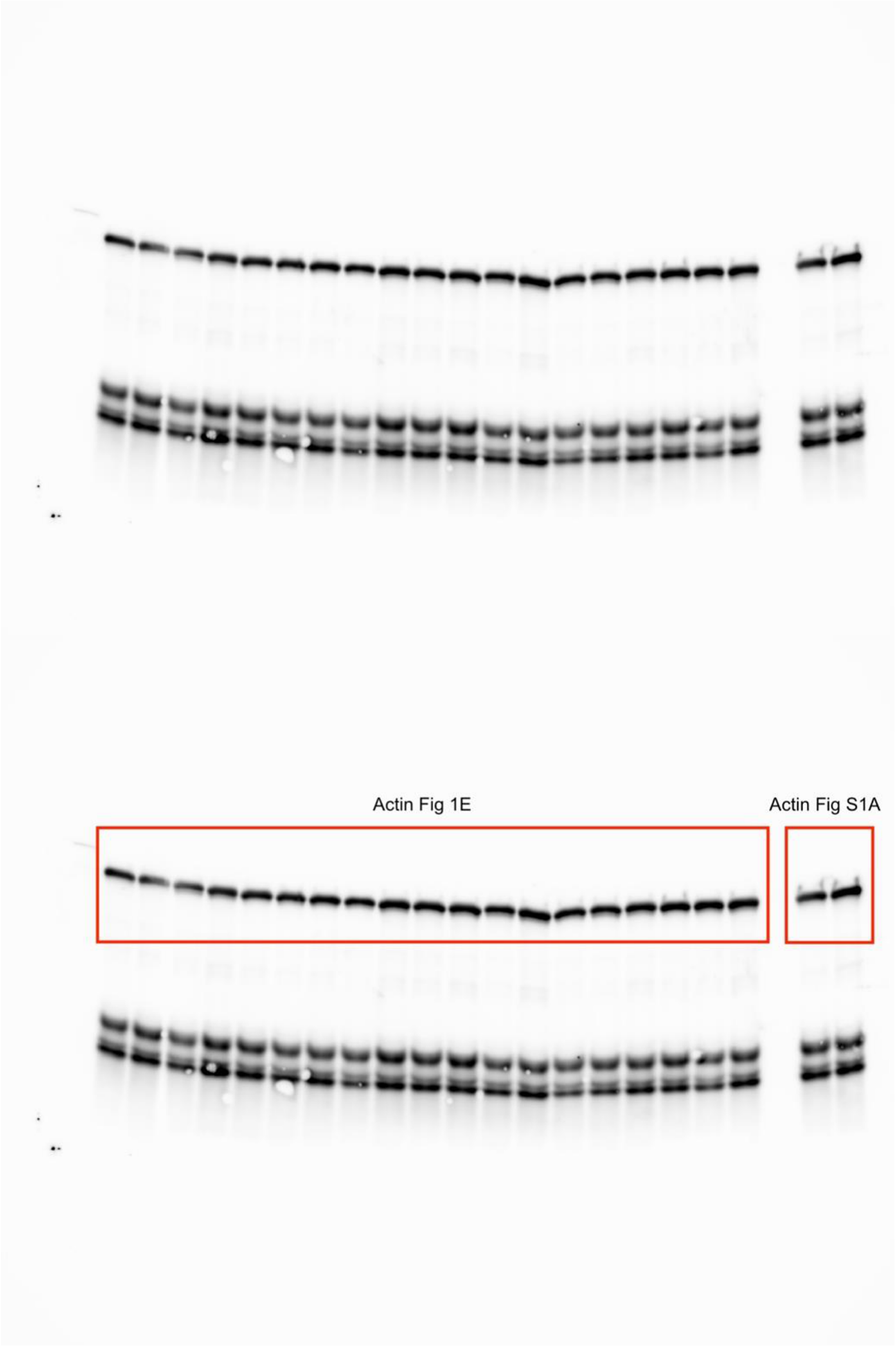

SARM1 Fig 1E and S1A

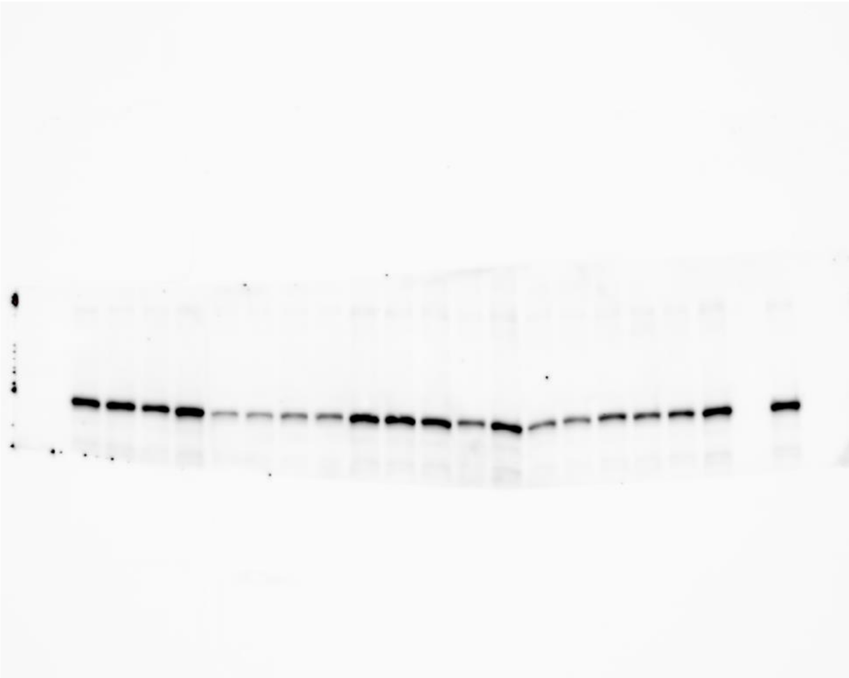

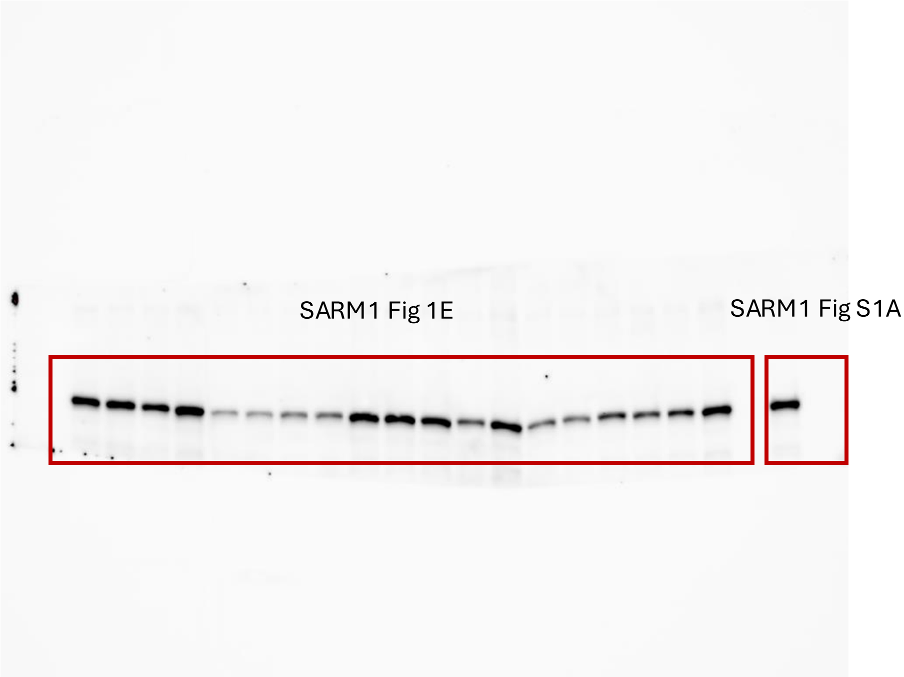

TH Fig 1E and S1A

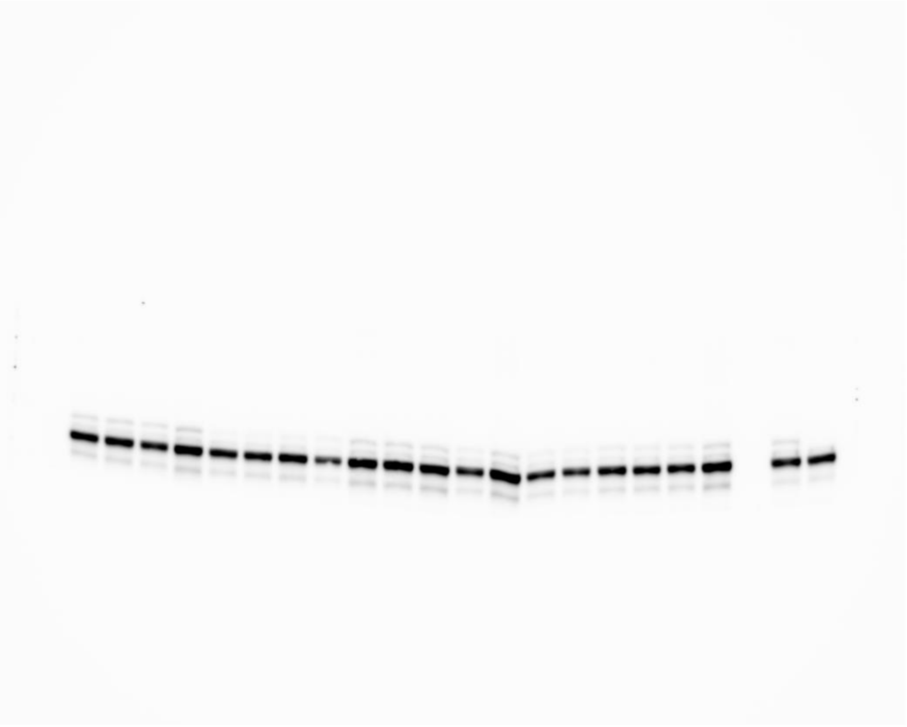

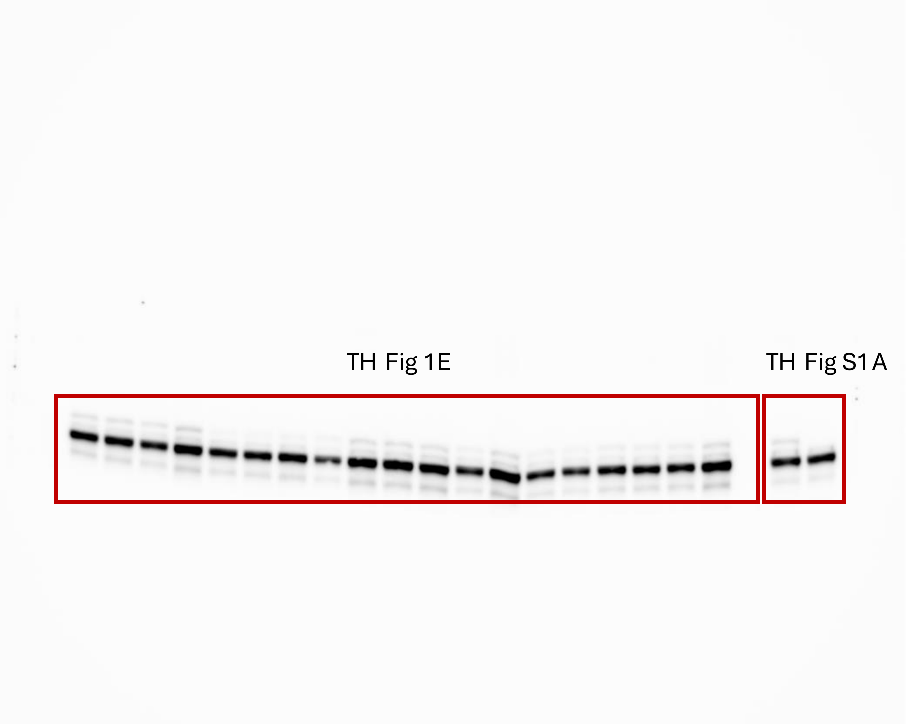

Actin Fig 1F

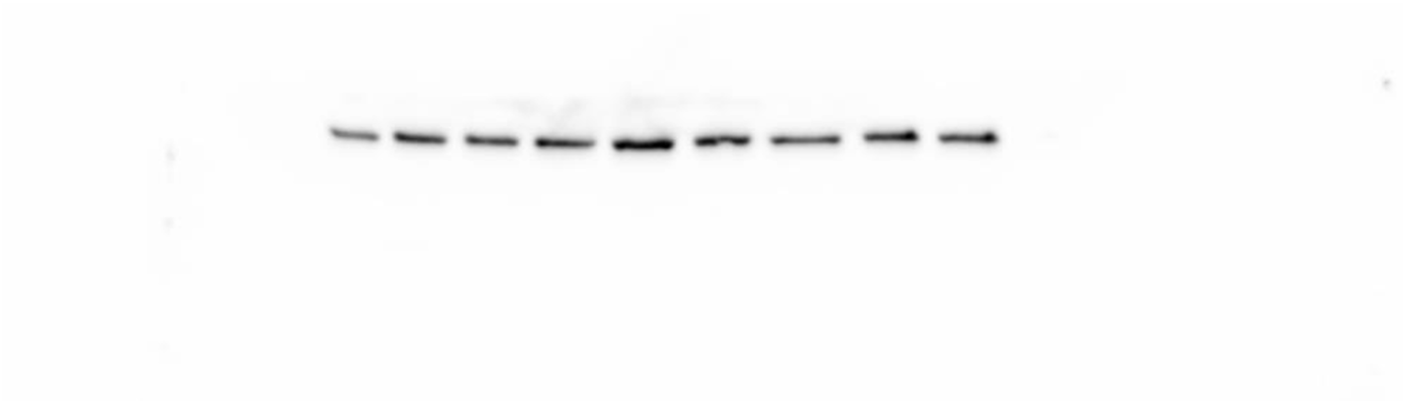

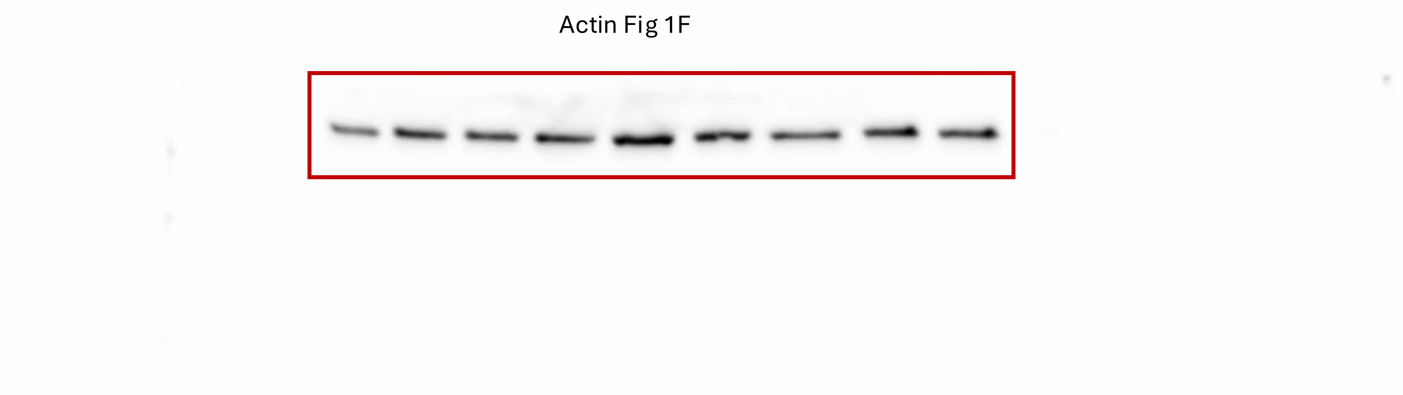

SARM1 Fig 1F

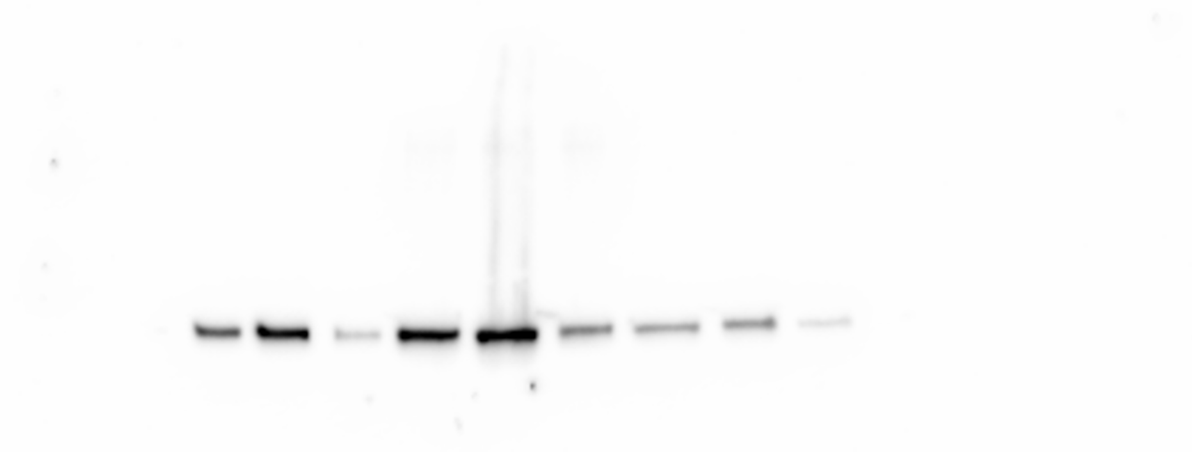

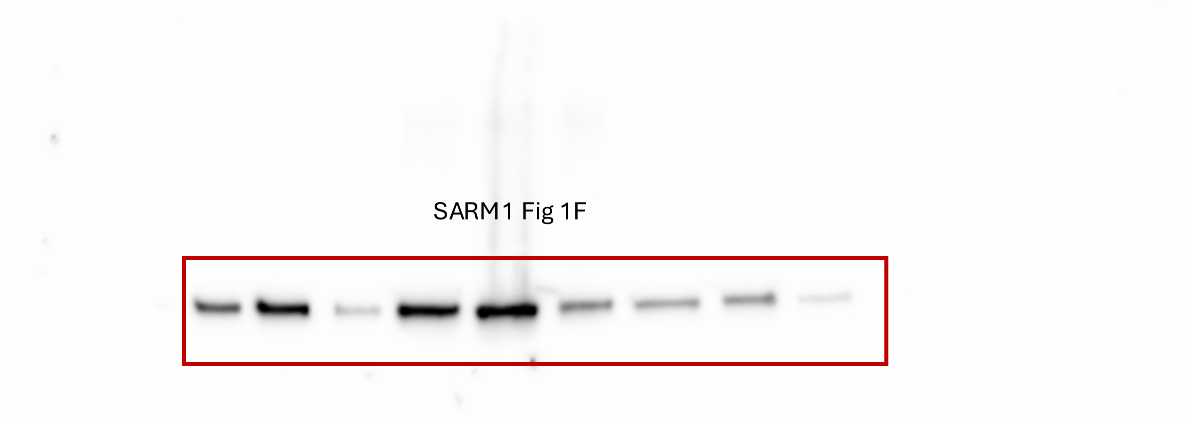

TH Fig 1F

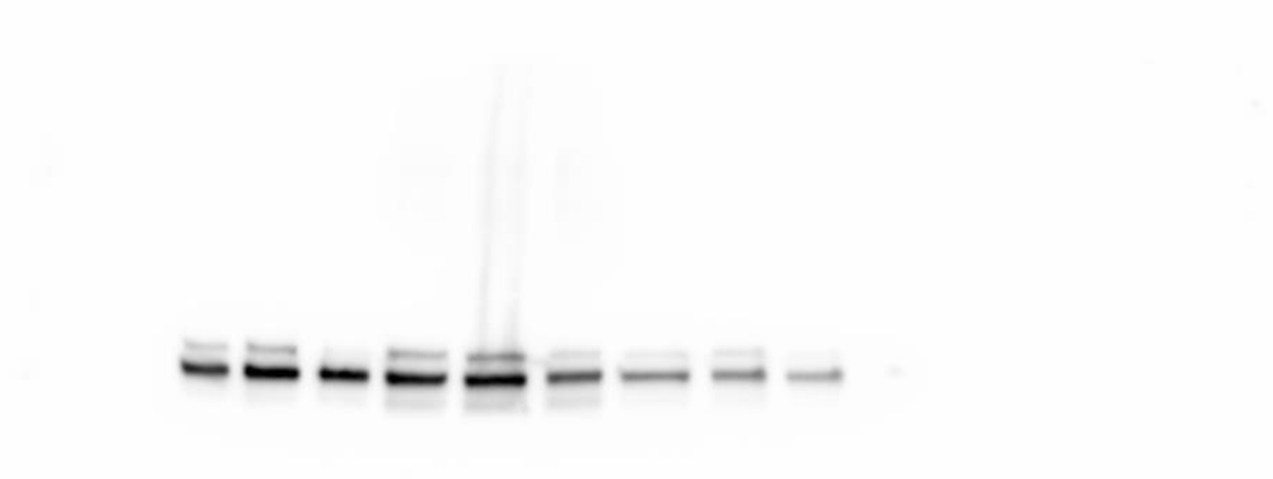

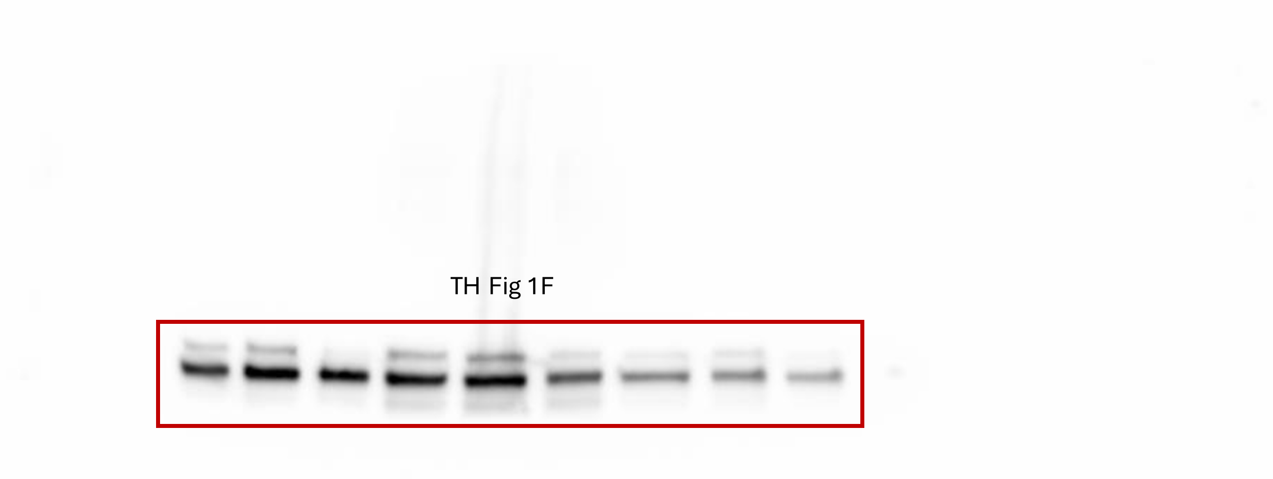

Supplement: Supplementary Material [file EMS207275-supplement-Supplementary_Material.pdf]
